# Research on the prediction model of mastitis in dairy cows based on time series characteristics

**DOI:** 10.3389/fvets.2025.1575525

**Published:** 2025-04-24

**Authors:** Rui Guo, Yongqiang Dai, Junjie Hu

**Affiliations:** ^1^College of Information Science and Technology, Gansu Agricultural University, Lanzhou, China; ^2^College of Veterinary Medicine, Gansu Agricultural University, Lanzhou, China

**Keywords:** time series prediction, somatic cell count, subclinical mastitis in dairy cows, XGBoost, SHAP value

## Abstract

**Introduction:**

Mastitis in dairy cows is a significant challenge faced by the global dairy industry, significantly affecting the quality and output of milk from dairy enterprises and causing them to suffer severe economic losses. With the increasing public concern over food safety and the rational use of antibiotics, how to identify cows at risk of disease early has become a key issue that needs to be urgently addressed. Especially subclinical mastitis, due to the lack of obvious external symptoms, makes detection more difficult, so early warning of it is particularly important.

**Methods:**

In this study, a time series prediction method, combined with machine learning techniques, was used to predict the risk of mastitis in dairy cows. The study data were obtained from the production records of 4000 dairy cows in a large farm in Hexi region of Gansu. By constructing time-series features, production indicators such as milk yield, fat rate and protein rate of each cow in two consecutive months, April and May, were utilized to predict its health status in June. To fully exploit the value of the time series features, we designed a multidimensional feature set that included raw indicator values, monthly change rates, and statistical features. After data preprocessing and sample balancing, data from 2821 cows were selected for model training. Finally, the applicability of each model was assessed by comparing and analyzing the prediction performance of six models, namely eXtreme Gradient Boosting(XGBoost), Gradient Boosting Decision Tree (GBDT), Support Vector Machine (SVM), K Nearest Neighbors (KNN), Logistic Regression, and Long Short-Term Memory Network (LSTM).

**Results:**

The XGBoost model demonstrated optimal performance, achieving an area under the ROC curve (AUC) of 0.75 with an accuracy rate of 71.36%. Feature importance analysis revealed three key temporal indicators significantly influencing prediction outcomes: May milk yield (22.29%), standard deviation of fat percentage (20.27%), and fat percentage change rate (19.87%). SHapley Additive exPlanations (SHAP) value analysis further validated the predictive value of these temporal features, providing dairy farm managers with clearly defined monitoring priorities.

**Discussion:**

The XGBoost model demonstrates strong potential as an accurate predictive tool for subclinical mastitis in dairy cows. This study presents an effective early-warning approach through time-series modeling that offers significant practical value for mastitis prevention in dairy farm management.

## 1 Introduction

Mastitis is one of the most common diseases in dairy cows, which not only significantly affects milk production and milk quality but is also a major cause of cow culling (1). With increasing concern about the use of antibiotics in animal husbandry, the early detection and prevention of mastitis without overreliance on antibiotic therapy have become a hot research topic (2). In particular, subclinical mastitis, which does not cause significant changes in udder appearance or milk quality, is mainly characterized by a significantly elevated somatic cell count (SCC) (usually more than 200,000 cells/mL) (3)—a feature that makes its diagnosis and early intervention more challenging.

In recent years, the Dairy Herd Improvement (DHI) assay system has gained widespread popularity in the global dairy farming industry due to the modernization of the dairy industry (4). The application of automated milking systems and routine milk testing procedures has accumulated a huge amount of production data for farms. These data include individualized metrics such as milk yield, milk fat percentage, and milk protein percentage as measured by DHI (5). The continuous and time-series characteristics of the data, along with the powerful data processing and pattern recognition capabilities of machine learning methods, have opened up new technological avenues for disease prediction and early warning in animal husbandry production (6). Among them, these technological tools have demonstrated distinct advantages in intelligent monitoring and early warning of mastitis, especially subclinical mastitis (7). In the detection of subclinical mastitis, diagnostic means have undergone an evolution from traditional manual detection to modern intelligent monitoring. Conventional diagnostic methods, such as SCC, microbial culture, and physical examination, are highly accurate but often require specialized personnel and are time-consuming. With the advancement of science and technology, monitoring technology has gradually developed in the direction of automation and intelligence. In recent years, novel detection techniques have emerged. Rodríguez-Hernández et al. (8) demonstrated good potential in the diagnosis of subclinical mastitis using near-infrared spectroscopy analysis. A predictive model was developed by analyzing milk samples from 101 cows, yielding classification accuracy ranging from 85.71 to 95.24%. This method is expected to serve as a rapid screening tool for occult mastitis in dairy cows and establish a basis for pathogen-based targeted therapy; however, the reliability of the model still needs improvement through increased sample diversity. Zhang et al. (9) proposed the CLE-UNet semantic segmentation algorithm to optimize the application of infrared thermography for diagnosing mastitis in dairy cows. By introducing the Efficient Channel Attention (ECA) mechanism, center-of-mass loss function, and ocular ellipse fitting operation, the algorithm achieved an Mean Intersection over Union (MioU) of 89.32%, and the accuracy, sensitivity, and *F*_1_-value in mastitis diagnosis were 86.67, 82.35, and 87.5%, respectively. However, these tests still face technical bottlenecks such as high false-positive rates, limited early warning capabilities, and relatively high testing costs. To overcome these limitations, researchers have started to explore the application of more comprehensive machine learning methods in predicting mastitis. Currently, commonly used algorithms include single models such as support vector machine (SVM) (10), logistic regression (LR), and K-nearest neighbor (KNN), as along with integrated learning models, such as random forest (RF) (11) and Gradient Boosting Decision Tree. Although machine learning methods show promising application prospects, there are still three major shortcomings in existing research. First, the mining depth of data temporal features is insufficient, making it difficult to fully use the temporal evolution features embedded in continuous monitoring data. Second, the predictive performance and generalization ability of the models need improvement, especially in the face of data from different farming environments, where the models exhibit unstable performance. Third, there is a lack of systematic analysis regarding the significance of features, which hinders the ability to provide more targeted management recommendations for production practices.

To address the above problems, this study constructs a multidimensional enlistment that includes raw features, rate-of-change features, and statistical features based on two consecutive months of production data from cattle farms. It also builds a prediction model using various machine learning algorithms, such as XGBoost (12), and thoroughly explains in depth the influence of features on prediction results through SHapley Additive exPlanations (SHAP) value analysis (13). The innovations of the study are mainly reflected in (1) making full use of the temporal characteristics of the data to construct a multilevel feature system; (2) proposing a more effective prediction scheme by comparing the performances of multiple machine learning algorithms; and (3) adopting the SHAP value analysis method, which improves the interpretability of the model. The aim of this study is to provide a reliable mastitis early warning tool for farms to facilitate early prevention of the disease.

## 2 Materials and methods

### 2.1 Initial dataset

The data for this study were obtained from some of the data in the dairy cattle performance measurement (DHI) of a large farm in the Hexi region of Gansu. This farm strictly adheres to industry standard operating procedures in terms of breeding facilities, feeding management, and disease prevention and control, providing a reliable and representative database for this study. In the initial stage, we selected the production records of 4,000 cows from April to May. Several key aspects of dairy production management were addressed. The basic information included cow identification numbers (CowID) for individual tracking, data collection dates recorded in “YYYY-MM-DD” format, and litter size information reflecting the reproductive history of the cows. In terms of production performance indicators, they include milk yield, which reflects the production level of cows, milk fat percentage, and milk protein percentage, which measure milk quality, and SCC, which is a key indicator of mastitis (14).

Data quality control is a crucial aspect of ensuring the reliability of research. For example, milk production or milk composition data for individual cows are not recorded on specific dates. If there are fewer missing values and the data are more evenly distributed, use the mean-fill method, which involves filling in the missing milk production data based on the average of the cow’s milk production on other dates. For missing data on some key data (e.g., SCC), if it is not possible to fill them using reasonable methods and the percentage of missing records is too high, the data records of the corresponding cows may be considered for deletion and processed to avoid misleading the model training. Outliers may exist in the data due to equipment failure, recording errors, or other accidental factors. For example, a cow’s milk production suddenly shows an extraordinarily high or low value, which is far from the cow’s usual milk production level. For these types of outliers, visualization methods (e.g., drawing a box-and-line plot) are used to identify the range of outliers initially. The reasonableness of the outliers is then determined based on the reality of the data and expertise. If it is not possible to correct the data accurately, the outlier data point can also be considered for deletion to ensure the accuracy and reasonableness of the data.

**Figure 1:**
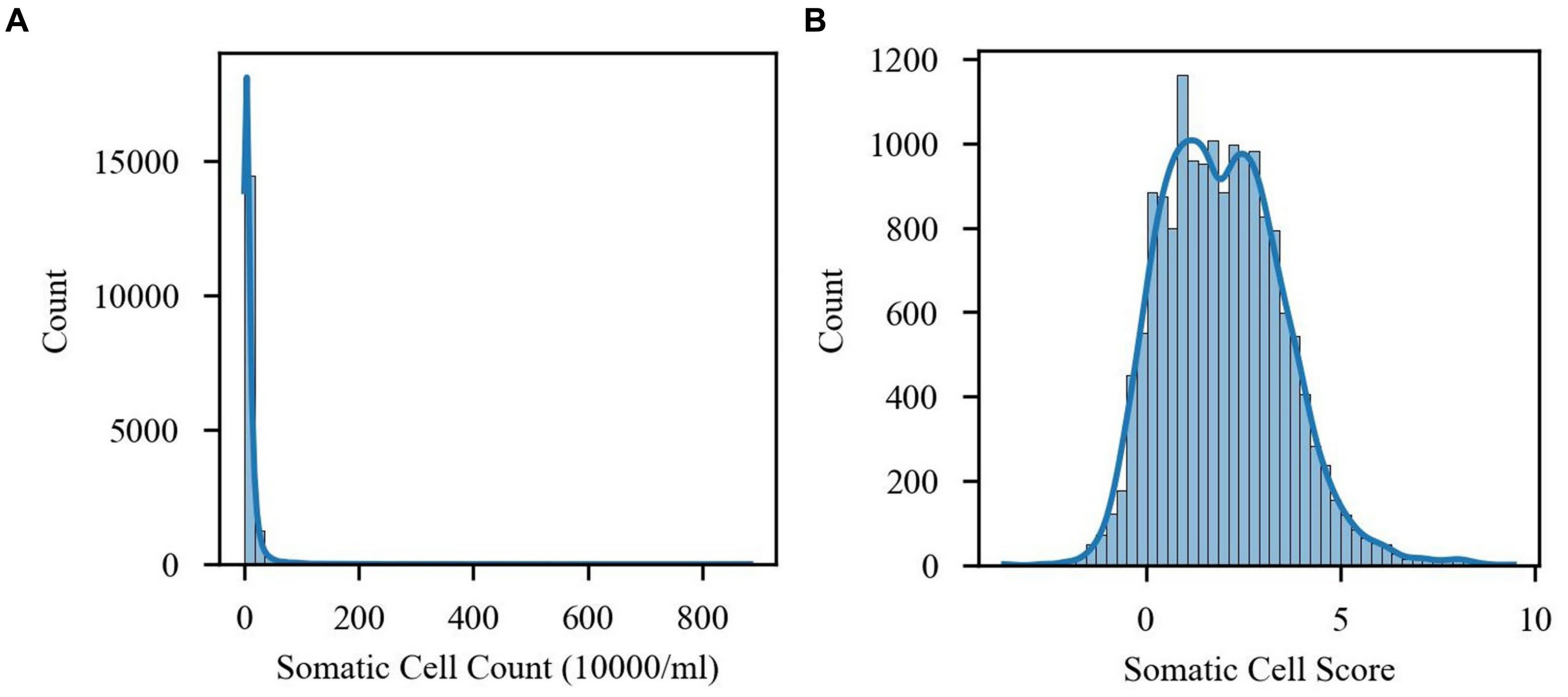
Comparison of the distribution of somatic cell count (SCC) and somatic cell fraction (SCS). **(A)** SCC distribution. **(B)** SCS distribution.

In the data preprocessing stage, considering that the SCC showed an obvious right-skewed distribution, the study converted the SCC to somatic cell score (SCS) with the conversion formula SCS = log_2_ (SCC/100,000) + 3 (15), which made the data distribution more resemble a normal distribution, as shown in Figure 1. This conversion not only helped the subsequent statistical analysis but also made the data more suitable for model training. This transformation not only helps subsequent statistical analysis but also makes the data more suitable for model training.

By analyzing the descriptive statistics of the preprocessed data, as shown in Table 1, it can be found that the sample herd as a whole showed better production performance and health conditions. In terms of milk quality indicators, the standard deviations of fat rate and protein percentage were 0.84 and 0.35%, respectively, indicating that the sample herd’s milk quality was relatively stable and fluctuated within normal limits. The median (29.6 kg) and the mean (29.66 kg) of the average daily milk yield were very close to each other with relatively small standard deviations, reflecting that the sample herds had a stable level of production performance. Although there was a large discrepancy between the maximum (69.6 kg) and the minimum (0.7 kg) values, this discrepancy mainly reflected the expected milk production characteristics of the different lactation stages. In terms of health status indicators, the distribution range of SCS (−3.64 to 9.46) was large, reflecting significant differences in individual health status in the herd. However, the interquartile range of SCC (22–98,000/mL) was mainly concentrated within the normal reference range, indicating good udder health in most cows. It is important to note that negative values of the converted SCS, which indicate very low raw SCC (<100,000/mL), typically indicate good udder health. The negative SCS values result from log transformation and do not indicate an actual negative SCC. These statistical characteristics not only reflect the representativeness and reliability of the sample data but also provide important basic data to support the subsequent development of disease prediction models.

**Table 1:** Descriptive statistics of dataset.

	Milk yield	Fat percentage	Protein percentage	SCC	SCS
Mean	29.66	4.67	3.78	9.91	1.94
Standard deviation	7.53	0.84	0.35	26.55	1.54
Minimum	0.7	1.41	2.46	0.1	−3.64
25%	24.8	4.1	3.53	2.2	0.81
50%	29.6	4.7	3.77	4.5	1.84
75%	34.4	5.19	4.03	9.8	2.97
Maximum	69.6	9.96	5.71	879.7	9.45

**Figure 2:**
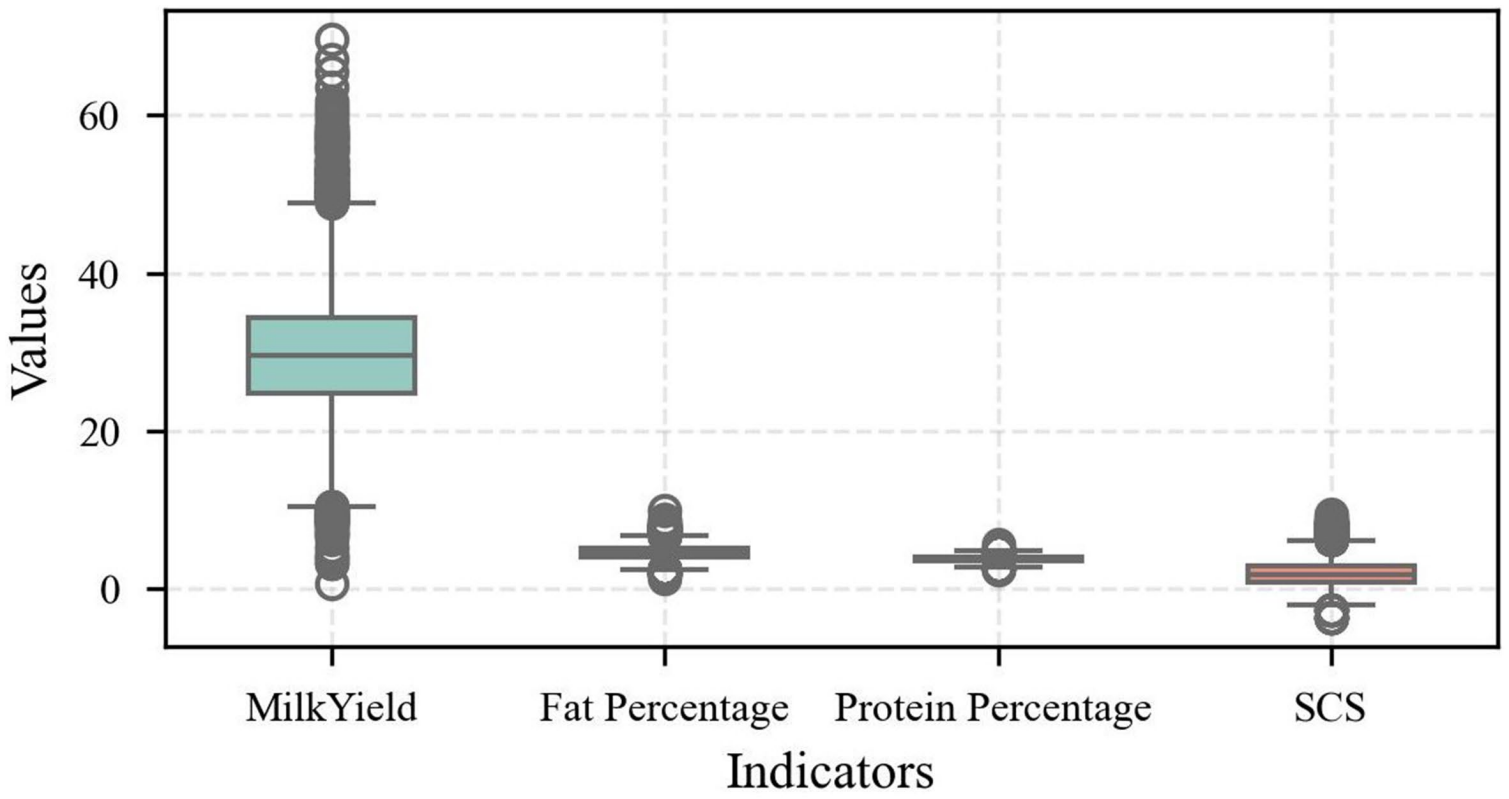
Distributional characteristics of the indicators.

This study uses box line plots (Figure 2) to depict milk yield, fat percentage, protein percentage, and SCS in order to more clearly illustrate the distributional properties of the indicators. It can be observed from the plots that milk yield showed large variability, with multiple upper outliers. This indicates that in the sample herds, a few cows’ milk yield was significantly higher than the group average. The difference in milk yield may be related to factors such as lactation stage and feeding management. In contrast, the relatively narrow bins for fat percentage and protein percentage indicate that these two indices remain relatively stable in the population, and this stability is important for maintaining milk quality. The distribution of SCS, on the other hand, showed a moderate degree of variability, and its outlier distribution characteristics reflected the presence of a small number of individuals with abnormal udder health status in the herd, which provides an essential reference for subsequent disease warning studies. Overall, the box plot not only visualizes the distribution of the indexes but also verifies the authenticity and reasonableness of the data, in which the observed “outliers” actually reflect the natural variability of the cow’s production performance, rather than errors in data collection or processing.

**Figure 3:**
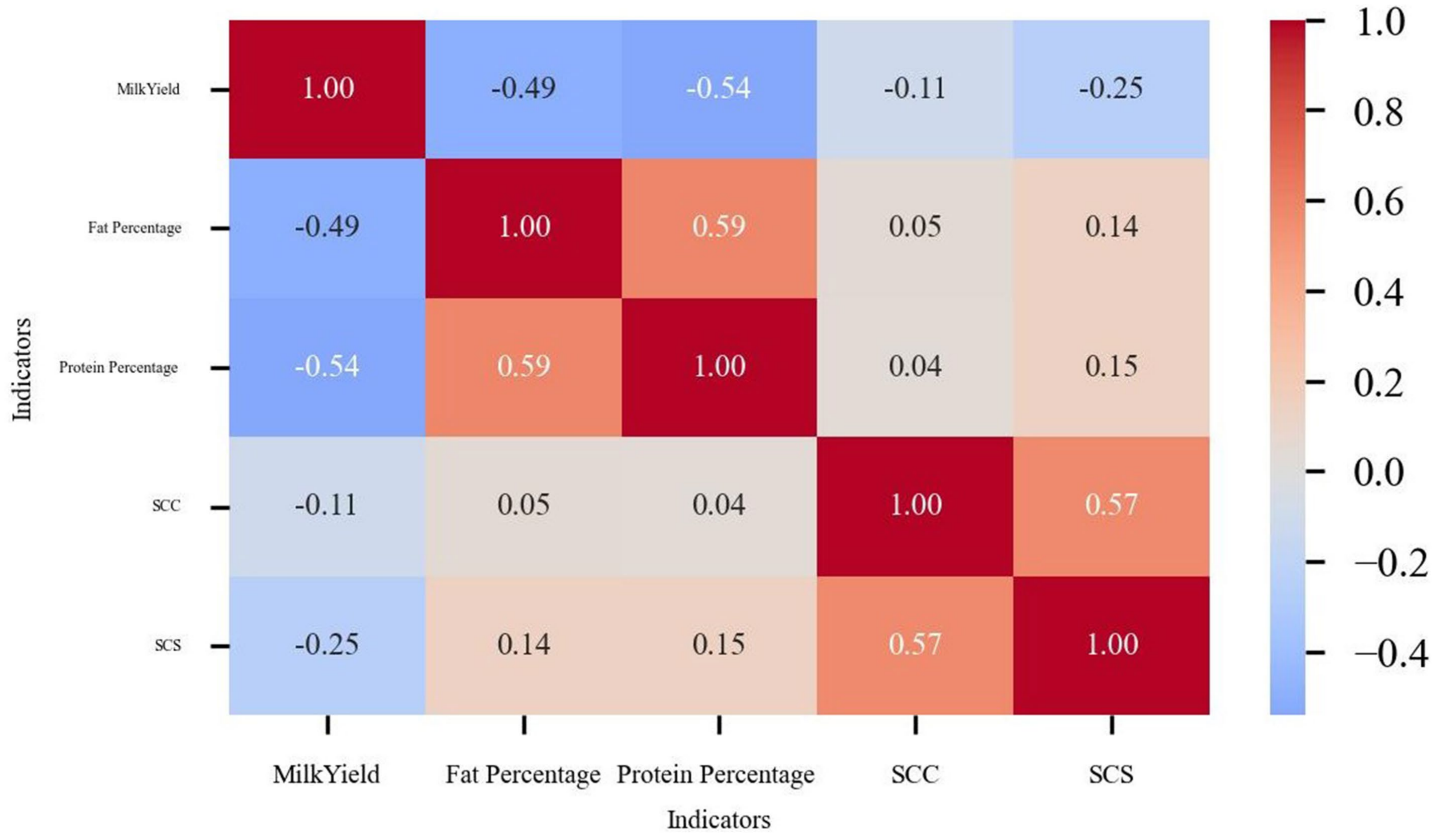
Indicator correlation heat map.

Additionally, a correlation heat map was used to analyze the data to identify the inherent relationship between the indicators (Figure 3). The color shades in the heat map visualize the strength of correlation between the variables. The data showed that milk yield was negatively correlated with SCC and SCS, which was in line with the biological law that udder health affects the milk production performance of cows. Meanwhile, the fat percentage and protein percentage showed a positive correlation with each other, which reflects the physiological correlation between dairy components. Notably, the strong correlation between SCC and SCS validated the data transformation.

To fully utilize the time-series characteristics of the data, the study systematically organized the data of three consecutive months, constructed monthly rate of change indicators reflecting the dynamic changes in production performance, and calculated statistical characteristics of the indicators, such as the mean and the standard deviation, to capture the long-term trend in the data and the fluctuation characteristics. These treatments laid a solid data foundation for the subsequent model construction.

The determination of udder health status in characterization studies primarily relies on somatic cell count (SCC) thresholds. According to international standards, SCC thresholds are categorized as follows: (1) 0–200,000 cells/mL for healthy status; (2) 200,000–500,000 cells/mL for subclinical mastitis; and (3) above 500,000 cells/mL for clinical mastitis. Given that no samples in our dataset exceeded SCC levels of 500,000 cells/mL, this study focused on the prediction of subclinical mastitis in cows with SCC values in the range of 0 to 500,000 cells/mL. Based on a previously validated SCC–SCS conversion model, where SCS = 4 corresponds to SCC = 200,000 cells/mL, we defined SCS ≥3 (equivalent to SCC ≥100,000 cells/mL) as being at risk for subclinical mastitis (label = 1), while SCS <3 was classified as healthy (label = 0). This threshold enables earlier disease intervention by detecting latent infection risks prior to reaching the diagnostic cutoff.

This study developed a comprehensive feature set comprising 13 predictive features, with the objective of effectively leveraging the temporal properties of the data in predictive feature construction. These features can be categorized into three main groups: first, raw features reflecting the basic production status of cows, including milk yield, milk fat percentage and protein percentage data for two consecutive months (April and May); second, rate-of-change features capturing the dynamic changes in production performance, including the monthly rate of change of milk yield, fat percentage and protein percentage; finally, statistical features reflecting the overall performance and stability, including the mean and standard deviation of milk yield and mean and standard deviation of fat percentage. The multilevel feature construction scheme aims to capture all aspects of cow health comprehensively, and these treatments lay a solid data foundation for subsequent model construction.

In terms of model selection, this study employed a variety of typical machine learning algorithms for comparative research. First, benchmark models representing different machine learning paradigms were selected: logistic regression as a representative of linear models, support vector machines (using the RBF kernel) based on kernel methods, the instance-based K-nearest neighbors algorithm (*K* = 5), Gradient Boosting Decision Tree (with 100 base learners) based on tree ensembles, and the XGBoost algorithm, which has shown excellent performance in structured data prediction tasks. The selection of these classical algorithms not only considered the diversity of model types but also took into account the differences in algorithmic complexity, thereby facilitating a comprehensive evaluation of the characteristics of the prediction task. However, most machine learning models have limitations in capturing the nonlinear features of real-world time series. In recent years, deep learning (DL) techniques have been successfully applied to time series prediction tasks, demonstrating significant advantages (16). To this end, this study also introduced the Long Short-Term Memory (LSTM) model to further explore complex patterns in time series data, providing new perspectives for enhancing predictive performance.

### 2.2 Experimental design

In this study, a systematic and rigorous methodology was used for the experimental design. First, to ensure the reliability of the model evaluation, a randomized division strategy was applied to the preprocessed production records of 2,821 cows, dividing the data into training and test sets in the ratio of 8:2. This division ratio ensured that there was sufficient data for model training, while retaining an appropriately sized test sample for independent assessment of model performance.

**Figure 4:**
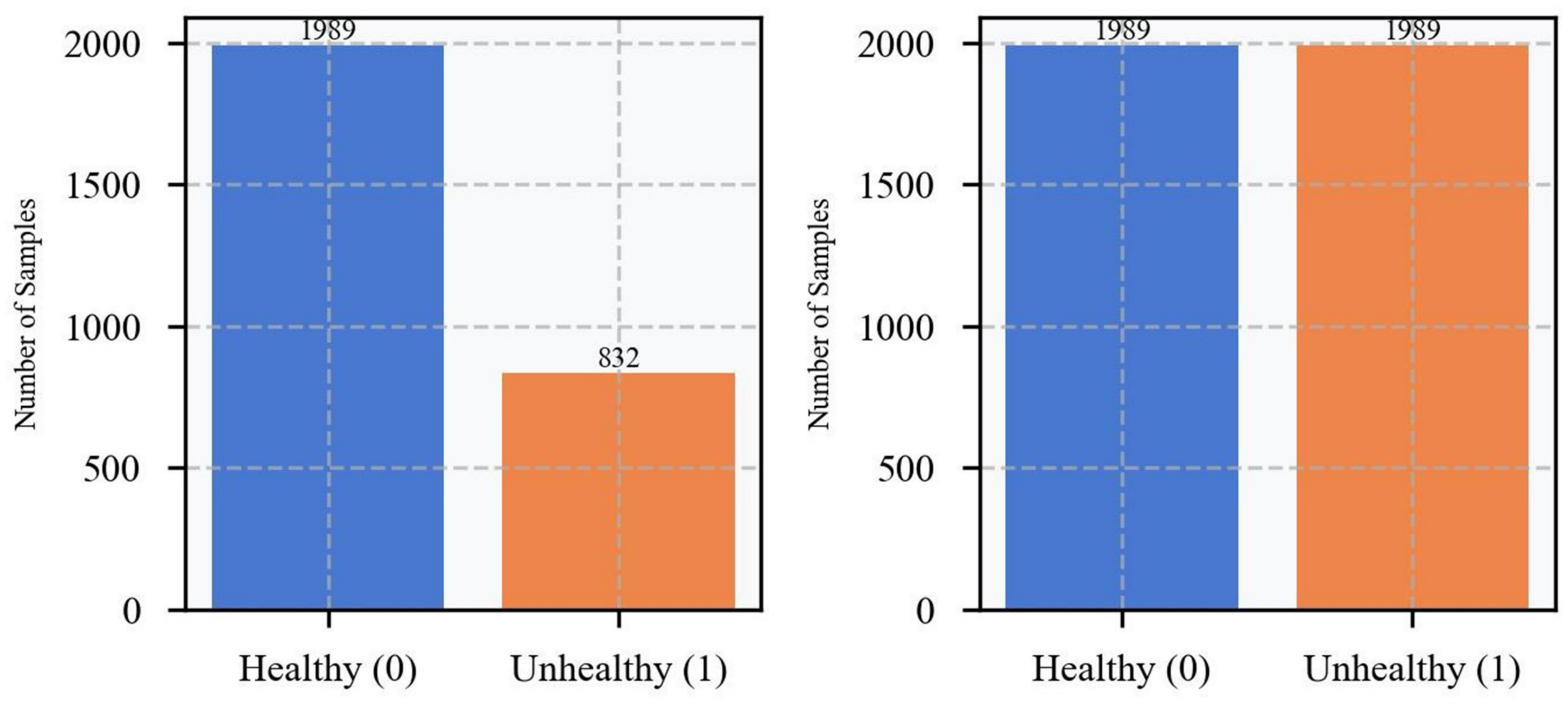
Before and after experiencing SMOTE sample distribution.

Prior to model training, the dataset was analyzed for category imbalance. As shown in the figure, there is a significant category imbalance in the original dataset, with approximately 2,000 healthy samples (labeled 0) and only approximately 800 unhealthy samples (labeled 1). Consequently, the number of healthy samples is approximately 2.5 times that of unhealthy samples. This significant class imbalance may cause the model to be overly biased toward the majority class during training, thus affecting the model’s prediction performance for the minority class. To address this issue, this study used the Synthetic Minority Oversampling Technique (SMOTE) to balance the data (17). The SMOTE technique increases the number of minority classes by generating synthetic samples in the feature space instead of merely replicating existing samples. As shown on the right-hand side of Figure 4, the number of unhealthy samples is boosted to approximately 2,000 after SMOTE processing, which is roughly equal to the number of healthy samples. This balanced processing method not only maintains the distribution characteristics of the original data but also effectively eliminates the category imbalance problem, creating more ideal data conditions for subsequent model training.

To comprehensively assess the performance and stability of the model, a 5-fold cross-validation (CV) method (18) was employed in this study. Specifically, the training dataset was randomly divided into five equal-sized subsets, with four subsets used for model training and the remaining subset used for model validation each time. This process was repeated for five iterations, and the average performance was then taken as the overall evaluation index of the model. During each iteration, the model is evaluated on different combinations of training and validation sets. This approach not only maximizes the utilization of limited data resources but also effectively evaluates the stability and generalization ability of the model. In the implementation process, AUC is used as an evaluation metric for each fold of validation, which is between 0 and 1. The closer the score is to 1, the better the performance of the model. By calculating the average AUC scores of the five validations, a more robust and reliable assessment of the model performance can be obtained. The advantages of this cross-validation method are that each sample is used as a training set and a validation set, which ensures the full utilization of the data; the impact of randomness brought about by a single division can be reduced by training and validating multiple times; the performance of the model on different data subsets can be effectively assessed, which can better reflect the generalization performance of the model; and the stability of the model performance can be assessed by analyzing the variance of the performance between different folds. In addition, to ensure the reproducibility of the experiments, a fixed random seed is set during the data division process, ensuring that the same data division results are obtained each time the experiment is run. Ultimately, the comprehensive performance of the model is not merely measured by the average AUC score, but a multidimensional evaluation system is adopted. Accuracy, as the most basic performance metric, intuitively reflects the overall judgmental accuracy of the model. *F*_1_-scores, as a reconciled average of precision and recall, provide a more comprehensive assessment of model performance. The combined use of these metrics ensures a thorough assessment of model performance (19).

For feature engineering, a sliding time window approach is used to integrate three consecutive months of data into a single forecasting unit. This design takes into account the temporal characteristics of the data, ensuring the real-time nature of the prediction. By calculating the rate of change and statistical characteristics of each index, the dynamic change characteristics of cow production performance were effectively captured.

A parameter optimization strategy combining grid search (20) and cross-validation was adopted during model training. By systematically exploring different combinations of parameters, such as the depth of the tree, the learning rate, and the minimum number of leaf node samples, the optimal model configuration was finally determined. Meanwhile, an early-stop strategy is used to avoid model overfitting by promptly stopping the training process when the performance of the validation set is no longer improving.

## 3 Results

### 3.1 Model performance comparison

**Figure 5:**
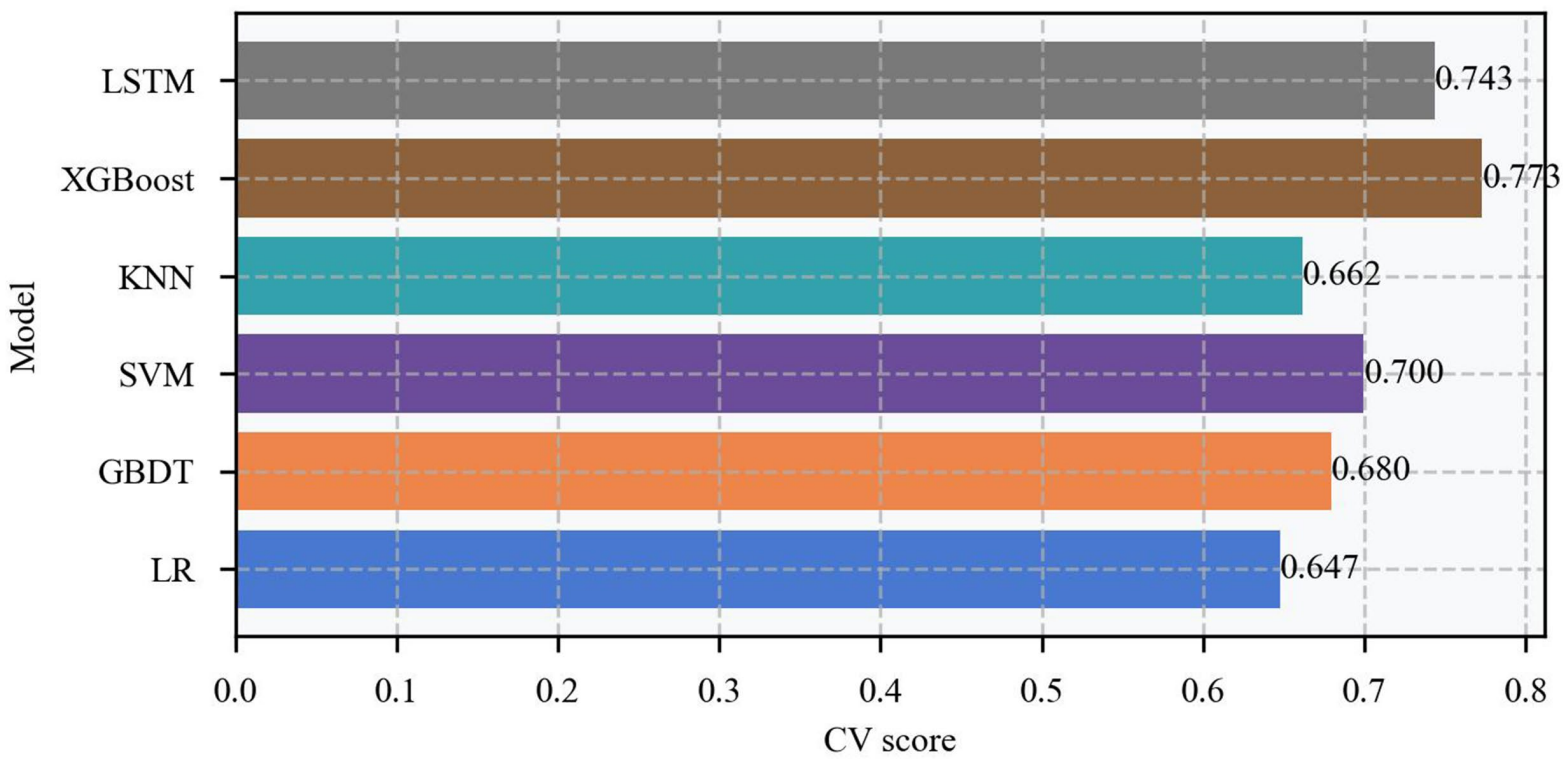
Cross-validation of different models.

This study systematically evaluated the predictive performance of six machine learning models, revealing significant differences in their predictive capabilities (Table 2). All key model performance metrics are provided with a 95% confidence interval. The XGBoost model demonstrated the best predictive performance, achieving an AUC of 0.75 and an accuracy of 0.71, outperforming other models across all evaluation metrics. Although deep learning models have significant advantages in time series prediction, the results indicate that the Long Short-Term Memory (LSTM) model also exhibited commendable performance. It was able to capture specific patterns in the time series data, achieving an accuracy of 65% and an AUC of 0.73. However, compared to the XGBoost model, which achieved an accuracy of 71% and an AUC of 0.75, the XGBoost model still outperformed the LSTM model. The Gradient Boosting Decision Tree (GBDT) model performed second best, with an AUC of 0.67 and an accuracy of 0.67. In contrast, the KNN (AUC of 0.64, accuracy of 0.65), SVM (AUC of 0.63, accuracy of 0.63), and logistic regression (AUC of 0.61, accuracy of 0.61) models showed relatively weaker performance. Based on the comprehensive evaluation metrics in Table 2, the XGBoost model maintained high levels across all indicators, including AUC, accuracy, precision, recall, and *F*_1_-score, demonstrating robust overall performance. To verify the stability of the model’s performance, the study used a five-fold cross-validation method for evaluation. As shown in Figure 5, the XGBoost model not only achieves the highest average performance but also exhibits the most stable performance across each fold, with receiver operating characteristic curve (ROC)-area under the curve (AUC) values consistently maintained between 0.72 and 0.78. This stability confirms that the model has good generalization ability and does not overly rely on specific training data. In contrast, while the other models also show some predictive ability, their performance fluctuates relatively more across folds, reflecting their limitations in handling complex biological prediction tasks. Although the gradient boosting tree model has the second-best overall performance, it is still less stable than the XGBoost model, which illustrates the unique advantage of XGBoost in handling the data of this study.

**Table 2:** Indicators for the evaluation of different models.

Model	AUC	Accuracy	Precision	Recall	*F*_1_-score
XGBoost	0.75 (0.72, 0.78)	0.71 (0.69–0.73)	0.71 (0.68–0.74)	0.71 (0.68–0.74)	0.71 (0.68–0.74)
LR	0.61 (0.58, 0.64)	0.61 (0.58–0.63)	0.62 (0.59–0.65)	0.62 (0.59–0.65)	0.62 (0.59–0.65)
GBDT	0.67 (0.64, 0.70)	0.67 (0.65–0.69)	0.67 (0.64–0.70)	0.67 (0.64–0.70)	0.67 (0.64–0.70)
SVM	0.69 (0.66, 0.71)	0.63 (0.60–0.66)	0.63 (0.60–0.66)	0.63 (0.60–0.66)	0.63 (0.60–0.66)
KNN	0.64 (0.61, 0.67)	0.65 (0.61, 0.68)	0.64 (0.61, 0.67)	0.64 (0.61, 0.67)	0.64 (0.61, 0.67)
LSTM	0.73 (0.70, 0.76)	0.65 (0.61, 0.68)	0.63 (0.60–0.66)	0.63 (0.60–0.66)	0.67 (0.64–0.70)

**Figure 6:**
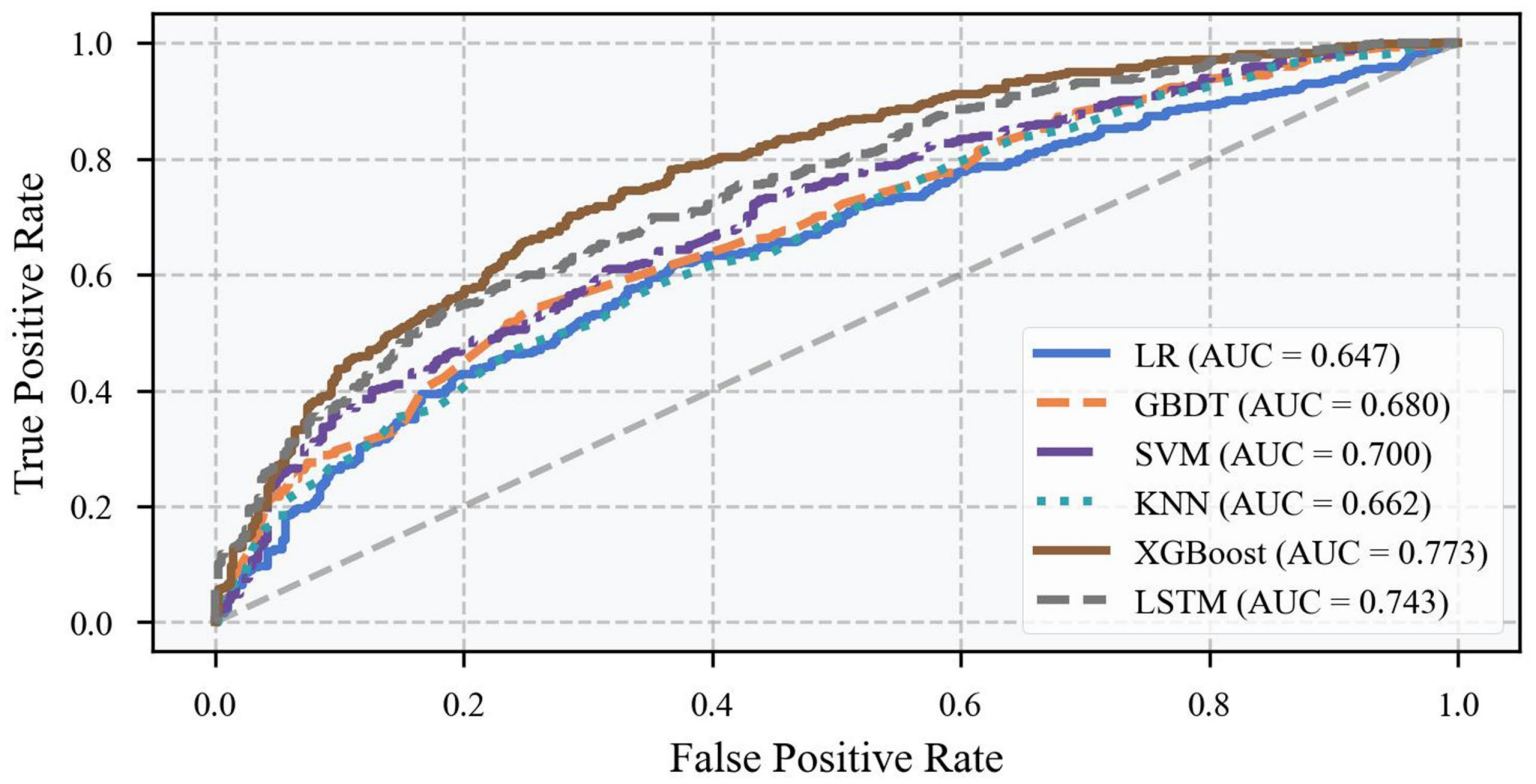
ROC curve for different models.

The performance of each model under different decision thresholds was further evaluated by ROC curve analysis (Figure 6). In the ROC curve graph, the horizontal axis indicates the false positive rate (1-specificity), and the vertical axis indicates the true positive rate (sensitivity). The closer the curve is to the upper left corner, the better the model performance. From the figure, it can be observed that the ROC curve of the XGBoost model is consistently located above the curves of the other models, and its AUC value reaches 0.77, which is significantly better than the other models. Especially in the middle part of the curve, the performance gap between the XGBoost model and other models is more prominent, which indicates that the model has a better ability to regulate the balance of sensitivity and specificity, and can flexibly adjust the prediction thresholds according to the needs of practical applications. The advantage of the ROC curve is of great significance for practical applications, because in disease prediction, we often need to adjust the sensitivity and specificity of the model according to the specific situation.

### 3.2 Analysis of the XGBoost model

Analyzing the classification effect of the XGBoost model in depth, we find that the model achieves a better balance in predicting both healthy and diseased samples. For both healthy samples (label 0) and diseased samples (label 1), the model achieved a precision of 0.71, a recall of 0.73 and 0.70, and *F*_1_-scores of 0.72 and 0.71, respectively. This well-balanced performance is extremely important for real-world applications, suggesting that the model neither over-alarms nor misses potentially diseased cases.

**Figure 7:**
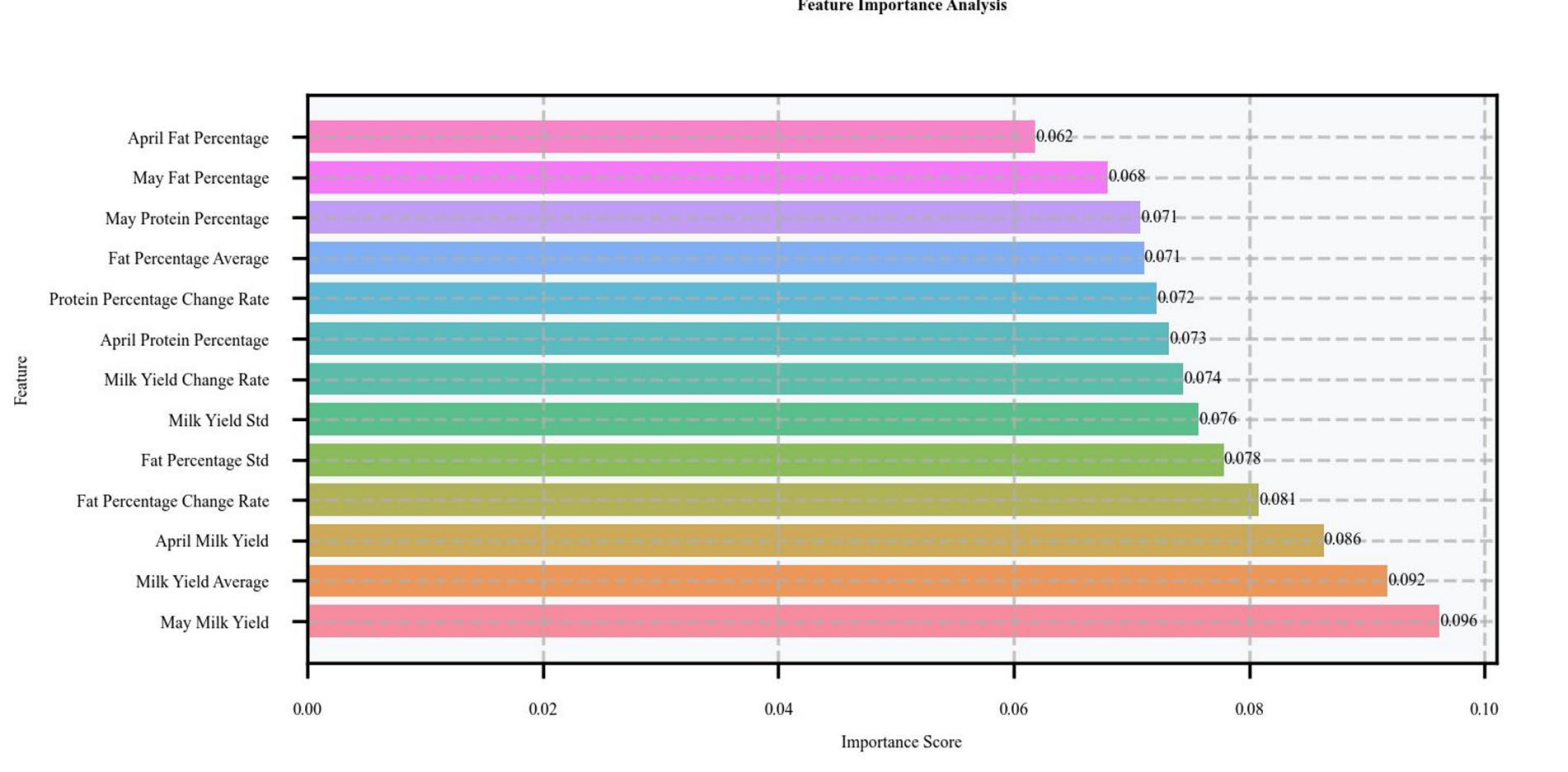
XGBoost feature importance analysis.

To understand the decision-making mechanism of the model, we performed a feature importance analysis based on SHAP values. This analysis revealed the order of contribution of each feature to the prediction results, as shown in Figure 7. May milk production, as the most important predictive feature, occupied an importance weight of 22.29%, indicating that the current month’s milk production performance had the most direct impact on predicting subclinical mastitis. Fat rate-related characteristics, including standard deviation (20.27%) and rate of change (19.87%), ranked second and third, indicating that the fluctuating characteristics of fat rate are key indicators of health status. In addition, the importance of April milk production (15.46%) and standard deviation of milk production (15.45%) should not be overlooked, as they reflect the vital contribution of historical milk production data and yield stability to prediction.

**Figure 8:**
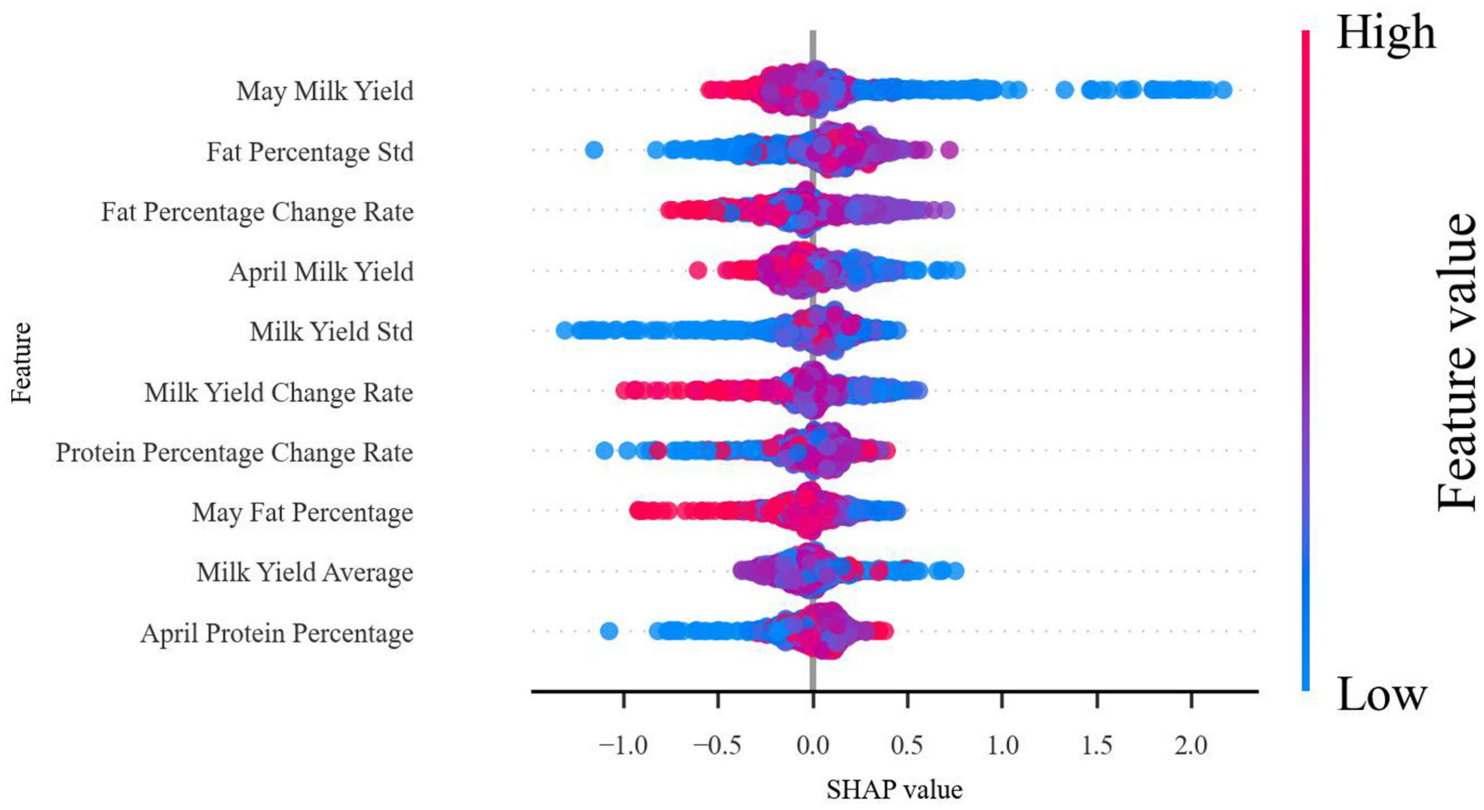
SHAP feature importance analysis.

Figure 8 illustrates the distribution of SHAP values, revealing the impact of various features on the prediction of subclinical mastitis. May milk yield emerges as the most significant predictive feature: high milk yield (represented by red dots) is generally associated with a healthy status, with positive SHAP values indicating that high milk yield reduces the risk of subclinical mastitis; conversely, low milk yield (represented by blue dots) is typically linked to health risks, with negative SHAP values suggesting that low milk yield increases the risk of subclinical mastitis. The standard deviation (Fat Rate Std.) and change rate (Fat Rate Change Rate) of fat percentage also demonstrate significant influence, as larger fluctuations are generally associated with an increased risk of disease. These findings provide specific monitoring directions for farm managers, such as tracking changes in milk yield and fat percentage to early identify potential health issues and implement intervention measures.

### 3.3 Timing characterization

Based on the above SHAP value analysis results, we can gain a deeper understanding of the role of time-series features in enhancing model performance. In this study, the predictive ability of the model was significantly enhanced by introducing the production data of two consecutive months (April and May) as predictive features. As can be seen from the analysis in Figures 7, 8, both May milk production (22.29%) and April milk production (15.46%) ranked among the top five in terms of importance, validating the value of continuous data in prediction. This application of time-series data allows the model to capture dynamic trends in cow performance rather than relying solely on static information at a single point in time, which is particularly important for predicting latent diseases such as subclinical mastitis.

When dairy cows experience mammary gland infections leading to subclinical mastitis, the immune system releases a variety of cytokines that disrupt the normal metabolic activities of mammary epithelial cells. This interference adversely affects the synthesis and secretion of milk components, such as fat and protein, resulting in significant alterations in milk yield and composition. These changes are prominently reflected in dynamic production indicators. The study revealed that the rate of change in fat percentage holds a critical predictive value, ranking third with an importance weight of 19.87%. As illustrated in the SHAP distribution in Figure 8, fluctuations in the rate of change in fat percentage exhibit a strong correlation with health risks, underscoring the importance of dynamic indicators in identifying potential health issues. Furthermore, the standard deviation of fat percentage ranked second with an importance weight of 20.27%, while the standard deviation of milk yield (15.45%) also featured among the top five most important indicators. These statistical metrics effectively capture the variability in production parameters, providing robust evidence for the early identification of cows at risk of disease. The SHAP value distribution plot further demonstrates that higher variability in these indicators is often associated with increased health risks, even when individual measurements fall within the normal range. These findings underscore the value of dynamic indicators in reflecting the immune system’s impact on mammary metabolism, offering a reliable approach for the early detection of subclinical mastitis.

The combined application of these three types of time-series features (consecutive month data, rate of change, and statistical features) forms a multilevel prediction system. From the results of the SHAP value analysis, this feature construction scheme not only improves the prediction accuracy of the model but also enhances the reliability and interpretability of the prediction results. From a practical point of view, the design of this feature system is also in line with the clinical experience of veterinary experts that performance and trends at multiple time points need to be considered together to assess the health status of cows.

## 4 Discussion

In this study, we have thoroughly discussed and analyzed the developed model for predicting subclinical mastitis. First, in terms of model prediction ability, the XGBoost-based prediction model achieved an accuracy of 71.36%, which is a more desirable result in complex biological prediction tasks. The results of the five-fold cross-validation show that the model’s performance remains stable across different datasets, with the AUC value consistently ranging from 0.72 to 0.78, which confirms the model’s good generalization ability. Meanwhile, the model achieves a good balance in predicting positive and negative samples, and the precision and recall of healthy and diseased samples are maintained at similar levels. This balanced classification performance is significant for practical applications.

The SHAP value analysis identified several important predictors in terms of feature importance. The trend of milk production as the most crucial predictive feature is reflected in the importance ranking of current-month milk production and historical milk production. The stability of fat percentage showed significant predictive value through its standard deviation feature, suggesting that the volatility of production metrics may be significant in determining health status. These findings not only improve the interpretability of the model but also provide specific guidance for practical production management.

In terms of practical application value, the predictive model developed in this study can serve as an effective early warning tool to help farms identify potential health problems promptly. By analyzing daily production data, the model provides early warning information before disease symptoms are evident, enabling to achieve accurate management and reduces detection costs. However, there are some limitations in the application of models. First, the effective operation of the model relies on continuous monitoring data, which puts high demands on the data collection and management system of the farm. Second, although the 71.36% prediction accuracy is of practical value, it still has room for improvement. In addition, the generalization performance of the model needs to be validated on a larger dataset comprising farms of varying sizes and management levels.

In light of these limitations, we propose several directions for future research to enhance the predictive models further. First, expanding the dimensionality of the data by incorporating additional types of production indicators, such as environmental parameters and feeding management data, could provide new predictive insights. Second, broadening the scope of data collection by integrating data from different regions and farms of varying scales could improve the adaptability and generalization capabilities of the models. On a methodological level, further optimization of feature engineering techniques, such as exploring intelligent optimization algorithms for feature extraction, is recommended. Remarkably, given the temporal nature of the data, adopting more advanced deep learning architectures like recurrent neural networks (RNNs) may lead to significant breakthroughs. Through these improvements, predictive models are expected to achieve greater accuracy and practicality, offering more robust technical support for dairy cow health management.

## 5 Conclusion

In this study, a prediction model for subclinical mastitis in dairy cows, based on the XGBoost algorithm, was successfully developed and achieved significant results in several aspects. The experimental results demonstrated that the model achieved a prediction accuracy of 71.36%, which is a significant improvement over traditional methods such as logistic regression. This performance improvement is not only reflected in the overall accuracy, but more importantly, the AUC value, which is crucial in practical applications, reaches 0.75, proving that the model has a strong differentiation ability.

To address data imbalance, which is a common challenge in the field of disease prediction, this study was effectively handled using the SMOTE method. This processing strategy enabled the model to achieve a more balanced performance in predicting both healthy and diseased samples, as reflected in similar precision and recall rates. This balanced prediction performance is important for practical applications, as it avoids both over-warning and reducing the risk of under-detection.

The time-series feature introduced in the study is one of the key factors for the success of the model. By constructing a multilevel feature system that includes consecutive-month data, rate of change, and statistical features, the model can effectively capture the dynamic change characteristics of cow production performance. The analysis of SHAP values shows that these time-series features play an important role in the prediction process, especially indicators such as milk yield, standard deviation of fat rate, and rate of change of the current month, which are ranked high, verifying the necessity and effectiveness.

The interpretability of the model is another important advantage. By analyzing the importance of the features, we clearly identified the key indicators that have the most significant influence on the prediction results. These findings are highly consistent with the clinical experience of veterinary experts, providing a clear guidance for the actual production management. This interpretability not only enhances the credibility of the model but also provides specific guidance for the refined management of farms. Overall, the prediction model developed in this study demonstrating promising application prospects and provides effective technical support for dairy farms to realize early warning of diseases and precise management.

## Data Availability

The data analyzed in this study is subject to the following licenses/restrictions: confidentiality: the data contains sensitive information that is not to be shared publicly due to privacy concerns related to the dairy farm operations and the individual cows’ performance metrics. Limited access: access to the dataset is restricted to the research team and authorized personnel only. Requests to access these datasets should be directed to dyq@gsau.edu.cn.
